# A Clinical, Biological, and Biomaterials Perspective into Tendon Injuries and Regeneration

**DOI:** 10.1089/ten.teb.2016.0181

**Published:** 2017-02-01

**Authors:** Grace Walden, Xin Liao, Simon Donell, Mike J. Raxworthy, Graham P. Riley, Aram Saeed

**Affiliations:** ^1^ School of Pharmacy, University of East Anglia, Norwich, United Kingdom.; ^2^ Norfolk and Norwich University Hospital, Norwich, United Kingdom.; ^3^ Norwich Medical School, University of East Anglia, Norwich, United Kingdom.; ^4^ Neotherix Limited, York, United Kingdom.; ^5^ University of Leeds, Leeds, United Kingdom.; ^6^ School of Biological Sciences, University of East Anglia, Norwich, United Kingdom.

**Keywords:** tendon injury, tissue engineering, tendinopathy, tendon rupture, injectable scaffold, implant

## Abstract

Tendon injury is common and debilitating, and it is associated with long-term pain and ineffective healing. It is estimated to afflict 25% of the adult population and is often a career-ending disease in athletes and racehorses. Tendon injury is associated with high morbidity, pain, and long-term suffering for the patient. Due to the low cellularity and vascularity of tendon tissue, once damage has occurred, the repair process is slow and inefficient, resulting in mechanically, structurally, and functionally inferior tissue. Current treatment options focus on pain management, often being palliative and temporary and ending in reduced function. Most treatments available do not address the underlying cause of the disease and, as such, are often ineffective with variable results. The need for an advanced therapeutic that addresses the underlying pathology is evident. Tissue engineering and regenerative medicine is an emerging field that is aimed at stimulating the body's own repair system to produce *de novo* tissue through the use of factors such as cells, proteins, and genes that are delivered by a biomaterial scaffold. Successful tissue engineering strategies for tendon regeneration should be built on a foundation of understanding of the molecular and cellular composition of healthy compared with damaged tendon, and the inherent differences seen in the tissue after disease. This article presents a comprehensive clinical, biological, and biomaterials insight into tendon tissue engineering and regeneration toward more advanced therapeutics.

## Introduction

**FIG. 1.**
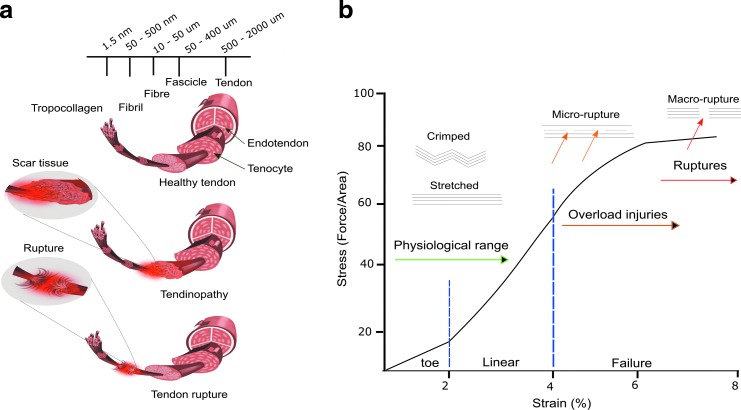
Showing tendon structures (physiological, tendinopathy, and tendon rupture) and stress–strain curve for tendon tissue. **(a)** Illustrating the tendon sub-structures (including fascicles, fibers, fibril, and tropocollagen), with relative dimensions thereof in a healthy tendon (*top-left*), and scar tissue formation in tendinopathy (*middle-left*)—characterized by disorganized collagen fibers, scar tissue formation, and tendon rupture (*bottom-left*) in which the two ends become separated and frayed. **(b)** A stress–strain curve for tendon tissue. At strains up to 2%, the tendon retains a characteristic crimped structure; this is known as the toe region. Under mild mechanical loads and stresses below 4%, the tissue is able to lengthen its crimped collagen fibers and withstand forces. This is known as the linear region, and it is representative of the physiological range of the tendon tissue. Strains above 4% can result in small micro-tears within the tissue, and tendinopathy can develop. Repeated micro-tears and strains above 8% can result in the tissue rupturing. The *blue dotted line* depicts the toe, linear and failure regions on the stress/strain curve.

Within the field of orthopedic surgery, damage to tendons is the most common soft tissue injury.^1^ Worldwide, of the 30 million musculoskeletal injuries reported, more than half are believed to involve tendons and ligaments.^2,3^ Tendon injuries predominantly affect the elderly population, and those involved with high mechanical load and weight-bearing activities such as athletes. It is believed that as many as 50% of sports-related injuries involve tendons.^4^ The prevalence of such injuries is only set to rise with the increase in average life expectancy and the popularity of high mechanical load activities such as gymnasium use, football, and athletics.^5^ Tendons are tough bands of fibrous, viscoelastic, connective tissue that anchor every muscle of the body to the bone, and they are responsible for resisting tension and aiding movement.^6^ It is critical that tendons have the ability to withstand large tensile forces exerted on them and are able to provide an efficient buffering system, absorbing shock and preventing muscular damage.^7^ However, this function also makes them susceptible to damage, microtrauma, and rupture. Tendons are able to withstand such forces by modifying their structure after stimulus via a process known as mechanical adaptation.^8^ At rest, a highly organized crimped configuration can be seen in the tissue that is conducive to the resistive capability of tendons.^7^ When under tensile strain, the crimped formation is one mechanism that enables the tissue to distend, absorbing large forces and acting as a buffer to shock. This conformational change is temporary and after the stimulus has receded, the tissue is able to revert to the characteristic crimped formation once again.^9^ The resistive capability of tendons is, however, not infinite, and if the stretching limit is exceeded, this formation is lost and the tissue becomes vulnerable to both micro- and macroscopic tears,^8^ as shown in Figure 1.

## Basic Tendon Anatomy

Tendons are formed from the continual aggregation of the smallest structural unit, collagen, into an increasingly complex architecture. Spontaneous aggregation of multiple collagen molecules results in the formation of collagen fibrils, which are aligned in a quarter-staggered array.^8^ These fibers then continually agglutinate to form progressive hierarchal structures beginning with a collagen fiber, leading to a primary fiber bundle, also known as a subfascicle, a secondary fiber bundle, termed a fascicle, a tertiary fiber bundle, and, ultimately, the tendon unit.^9–11^ This structure is highlighted in Figure 1.

Collagen type I accounts for 95% of total collagen in the tendon,^8^ and for 60% of the total dry mass of the tissue.^7^ Highly organized and aligned, with stable crosslinks throughout,^12^ it is representative of the mature form of collagen fibrils.^13^ The highly organized packing of collagen type I and hierarchical architecture confer on tendons the ability to stretch on mechanical stimuli and to absorb the resulting forces, enabling locomotion.^10^ The tenocytes present in tendon tissue have the ability to respond to stimuli and are able to remodel its microenvironment in response to stresses and strains. This is mediated by the action of matrix metalloproteinase and is essential for the repair, development, and function of the tendon tissue.^12^ Comprising proteoglycans, glycosaminoglycans, glycoproteins, and collagens,^8,14^ the extra cellular matrix (ECM) has a multitude of functions. By retaining large amounts of water, the ECM acts as a lubricant to surrounding tissues, ensuring the easy gliding of fibers during mechanical deformation.^5,9^ In addition, it confers elasticity, provides cell adhesion sites, and binds secreted growth factors.^14^

## Tendon Repair and Regeneration

The repair of tendon tissue is hindered by its inherit low cellularity and metabolic activity.^11^ The predominant cell type found in tendon tissue is the elongated fibroblast-like cell that is known as the tenocyte.^12^ Widely used markers for tenocyte characterization such as Smad, scleraxis, tenomodulin, and collagen are nonspecific and are expressed in most cells present within tendon tissue. This makes their characterization extremely difficult and, thus, hampers the clinical translation of *in vitro* results. In addition, tendon stem cells (TSC) express a multitude of markers that are also found in both mesenchymal and embryonic stem cells.^15^ It has been suggested that to fully characterize tenocytes, cells must exhibit a combination of spindle-shaped morphology, combined with positive expression of markers such as collagens, decorin, scleraxis, and Tenomodulin, while also being negative for chondrogenic, osteogenic, and adipogenic markers. Currently, more work is needed to find this ideal panel of markers for tendon-specific cell characterization.^2,16^

Tenocytes account for as much as 95% of the cellular composition of the tissue^7,10^ and are situated within the aligned collagen fiber bundles and the epitenon and endotenon.^12,17^ These cells are responsible for the synthesis of collagen, essential for the hierarchical architecture of the tissue, and extracellular matrix components.^11,18^ The further 5–10% of cellular composition comprises progenitor cells, chondrocytes that are necessary for enthesis formation, synovial cells, and vascular cells, including smooth muscle and endothelial cells that are needed for blood vessel development.^10,12^ Once tendons have been subjected to excessive strain, the healing process is initiated. Currently, two mechanisms of healing, intrinsic and extrinsic, are believed to occur after injury.^11^ Most repair is believed to be via the intrinsic pathway where proliferation of fibroblasts from the epitenon and endotenon occurs, resulting in cellular migration to the site of the lesion and the synthesis of new matrix materials.^11,19^ The extrinsic pathway is associated with migratory inflammatory cells and fibroblasts from surrounding tissues.^11,19^

The healing process consists of three overlapping phases: immediately after injury when hematoma formation and an inflammatory response occurs, followed by a repair process characterized by an increase in myofibroblasts and synthesis of new matrix materials, and, finally, a maturation and remodeling stage.^19^ In tendons, this remodeling process can last up to 1 year, and it results in a structurally and biomechanically inferior tissue that is characterized by disorganized, aberrantly aligned collagen and the presence of scar tissue.^20^ Healed tendon tissue is no longer comparable to its native form and the random alignment of synthesized collagen fibers, the disorganized matrix, and an increase in collagen type III lead to impaired function of the tissue.^7,10,21,22^ For the patient, this results in a reduction of mobility^23^ and an increase in pain and morbidity. Overall, the tendon is weaker and prone to rupture and tears,^19^ making it more likely for the clinical condition, known as tendinopathy, to develop.

## Tendinopathy

Tendinopathy is the umbrella term used to describe a broad spectrum of several different tendon pathologies^24^ resulting from overuse and excessive mechanical loading, preventing the tissue from being able to withstand further tension.^8,25^ Most tendinopathies are not caused by one single factor, and there are many contributory elements that can be either intrinsic or extrinsic.^26^ These can include age, gender, disease, occupation, and physical training.^10,26^ Tendinopathy can be classified as either acute, resulting from excessive overload, or chronic, due to a degenerative condition that is persistent over time.^14^ This explains the tendency for tendinopathy to occur in both young patients with active lifestyles and the elderly.^27^ Degeneration and consequently repeated microtrauma are considered the primary causes of chronic pain-free tendinopathy. As a result of continual tearing and failed healing attempts, the tissue that remains is weaker and at risk of “spontaneously” rupturing.^20,26^ It has been suggested that in as many as 97% of spontaneous ruptures, an underlining degenerative pathology was pre-existing before the incident^28^

The tissue formed after tendon injury is altered in both its structure and its composition. Degenerative tendinopathy is associated with a decrease in collage type I, which is indicative of a weakened tendon and results in decreased tensile strength and ability to withstand mechanical load.^29^ In addition, injured tendons exhibit increased expression of collagen type III. Collagen type III has fewer crosslinks^19,30^ and fibers that are smaller and thinner compared with collagen type I, with a decreased resistive capacity.^31^ Tendon tissue produced after healing, abundant in Collagen type III, is much less organized, which is believed to be attributable to a loss of structure and a decreased mechanical strength.^8^ In healthy tendon, it is synthesized at significantly lower levels in comparison to collagen type I.^30^ However, in aging, degenerative, or stressed tendon, it is upregulated and synthesized in abundance during the repair process.^13,30^ An increase in the production of collagen type III, relative to type I, is believed to lead to the formation of adhesion sites. Adhesion sites result in inadequate lubrication between the tendon and surrounding tissues, causing friction and pain.^32^ These hinder the gliding of the tendon and account for its reduced mobility seen after injury.^33,34^ Increases in collagen type III are also associated with reduced mechanical strength, risk of rupture, and, ultimately, the formation of scar tissue.^30^ Likewise, tendinopathies are characterized by a broad range of cellular and molecular differences from the native tissue.^12^ Decreased cellularity is prominent, resulting from apoptosis of tenocytes.^35^ Conversely, tendinopathy can also exhibit hypercellularity,^36^ particularly in the case of tendinosis and chronic tendon pain. On injury, the cells can respond to intrinsic and extrinsic signals by increasing proliferation. These cells are often altered in morphology when compared with healthy tenocytes.^37^ Matrix organization is aberrant. Abnormal vascularity is present; the protein content and composition is altered, with an increase in tenascin-C and fibronectin and a decrease in decorin expression.^19^

Current treatment options for tendon injuries are mainly conservative, focused on the management of pain rather than on the healing of the underlying damage.^12^ Nonsteroidal anti-inflammatory drugs such as ibuprofen are used, where inflammation is a key component, for example, tendinitis. Exercise and mobilization therapy is generally recognized to be of benefit, with stretching and strengthening activities being the most commonplace.^38^ Surgery is considered the last resort and is used for the treatment of tendinopathy when conservative therapy has failed.^39^ Ruptures resulting in lesions greater than 5 mm have limited healing capacity and result in loss of function of tissue, meaning that surgery is usually the only option, notably in fit and active patients. Surgical treatment aims at bringing the repaired tendon back to its original length by excising the necrotic tissue and suturing the ends together.^40^ The remaining tendon, however, is often plastically deformed and therefore not as mechanically strong as the normal tendon. However, re-rupture is common in many affected tendons; in the rotator cuff, it has been reported to be as high as 94%.^22^ Long-term outcomes for patients are highly variable,^19^ and morbidity remains high even after so-called “successful” treatment.^11^ In addition, further complications postoperatively are prevalent, such as the increased risk of early onset osteoarthritis, chronic pain, and in the case of allografts, the risk of immune rejection. Surgery is yet to yield consistently satisfying results without the presence of pain, reduced patient mobility, morbidity, or high risk of re-rupture for patients.^39^ Overall, there is no accepted standard treatment option for tendon injuries, and there is an obvious unmet clinical need. The current therapies are not supported by satisfactory clinical trials, are not effective,^14^ and fail to address the underlying associated pathophysiological pathways.^27^ When considering the current treatment options available, the need for the development of a successful system that incorporates the knowledge of the native tissue and that aims at addressing the underlying pathological issue with the goal of a regenerative outcome is apparent.^8^

## Biological and Biomaterials Approaches in Tendon Regeneration

The emerging field of Tissue Engineering and Regenerative Medicine is increasingly being employed to design strategies for the repair of tendon tissue. These strategies are focused on the activation and enhancement of the body's own repair system by using a combinatory approach that may include the application of cells, stimulatory factors, genes, and scaffolds.^41^ Tissue engineering is evolving toward the production of functional *ex vivo* tissue manipulating the use of bio-responsive scaffolds,^42,43^ which can be implanted at the injury site, for *in situ* formation of *de novo* tissue.^44^ Recent attempts have been made both *in vivo* and *in vitro* to assess the advantages of delivering cells, genes, and proteins to tendon defects to recapitulate the complex signaling process seen in the natural healing of tendon tissue.^23^

## Cell-Based Therapy in Tendon Regeneration

One idea recently emerging in the field of tissue engineering is that the delivery of cells that are capable of synthesizing matrix materials may prove effective for the healing of tendon tissue.^45^ Different cell sources have been investigated, including tissue-specific cells such as tenocytes,^35,32^ and TSC^2,46^ as well as “nonspecific” mesenchymal cells^11,17,33,47,48^ derived from multiple sources, including bone marrow and adipose tissue. Direct injection of cells is proving promising, with several clinical trials underway. In one trial, an autologous tenocyte injection into the site of severe tendinopathy of the common extensor tendon in 20 patients that was associated with chronic lateral epicondylitis was performed.^35^ Overall, the outcome was one of improved function and improved repair of the tendon tissue. Patients reported an 86% improvement (i.e., reduction) in pain after treatment. Similarly, results from tests aimed at assessing the disabilities of the arm, shoulder, and hand improved by 91% compared with preintervention scores. In addition, magnetic resonance imaging (MRI) data aimed at grading the severity of tendinopathy showed a decrease in severity after treatment.^35^ However, this study used only a small sample size, with no control group and the level of evidence is four, which is deemed low. In addition, improvements were seen when comparing patient data based on the opinions of the patient rather than on histological examination of the injury. In fact, MRI data showed that in some patients the injury had worsened and needed further treatment. Dermal fibroblasts, an abundant and readily accessible cell source, have also been used in therapies for tendon regeneration.^49^ Twelve patients diagnosed with refractory lateral epicondylitis were treated with laboratory-prepared cells derived from dermal fibroblasts. After 6 months, a reduction was exhibited in the number of tears, angiogenesis, and tendon thickness. Recovery in 11 of the patients treated was deemed satisfactory, with ultrasonography results indicating the presence of tendon tissue resembling the native state.^50^ Forty-six patients suffering from refractory patellar tendinopathy were treated with ultrasound-guided injections of autologous skin-derived cells. Pain, severity of tendinopathy, and functional disability scores were measured; these improved from 44 to 75 points at 6 months post-treatment, which was consistent with a reduction in disability and pain for the patient.^49^

Preclinical animal studies have also shown promising results. Autologous tenocyte preparations were injected into artificial collagenase-induced chronic tendinopathy in rabbit Achilles. Production of collagen type I but not type III was increased, and these fibers were highly organized and aligned. The tissue produced had a tendency to be stiffer when compared with nontreated control groups.^51^ In equine models, bone marrow-derived mesenchymal stem cell (BM-MSC) treatment has resulted in a quicker recovery time and a reduction in re-injury rates.^52,53^ Autologous BM-MSCs suspended in bone marrow supernatant were injected into damaged digital flexor tendons of 12 race horses. Treated animals displayed improved tendon stiffness, and a return of characteristic crimp formation of tissue along with a recovered organization. Improved histological scores were recorded, and these were accompanied by decreased vascularity, water content, swelling, and MMP-13 activity. The cross-sectional area of tendons remained significantly smaller than control groups treated with saline alone. Overall, repaired tendons showed histological and biomechanical properties that were more similar to those of undamaged tendons when compared with untreated groups.^53^

Similarly, racehorses considered to have career-ending tendinopathy showed improved healing when treated with BM-MSC. An intralesional injection for naturally occurring tendinopathy of the digital flexor tendons was administered in 113 racehorses. After 3 years, 98.2% had returned to racing and re-injury rates were lower than with conservative treatments alone. This study concluded after long-term follow-up that the implantation of BM-MSC was both safe and effective for the treatment of tendinopathy.^52^

Combinatorial approaches have been investigated for the delivery of cells and proteins simultaneously. When injected in combination with platelet-rich plasma (PRP), adipose-derived stem cells (ADSC) led to the production of neo-tendon that was comparable to healthy tissue. Alignment of tendon fibers and significant reductions in lesion size were reported. Horses treated in this manner were able to return to competitive activity, and ultrasound data indicated the formation of neo-tissue that was comparable morphologically to healthy tendon, suggesting the initiation of a regenerative healing process.^54^ Current advances in cell-based therapy for tendon tissue regeneration are highlighted in Table 1.

**Table 1.** Current Advances in Cell-Based Strategies for the Regeneration of Tendon Tissue

*Cell line*	*Tendon model*	*Results*	*Type of study*	*Ref.*
BM-MSC	Equine	Improved tissue organization. Formation of crimp structure. Histological improvement of tissue, including reduction in GAG, DNA, and cell content, comparable to “normal” tendon.	*In vivo*/*in vitro*	^53^
ADSC	Rabbit Achilles tendon	Neo-tendon formed, with tensile strength comparable to 60% of normal tendon. Production of parallel collagen fibers and elongated cells aligned longitudinally with collagen fibers.	*In vivo*/*in vitro*	^55^
ADSC	Rabbit Achilles tendon	Increased tensile strength of tendon tissue. Partially regular and longitudinal alignment of collagen fibers. Increased collagen type I production.	*In vivo*/*in vitro*	^29^
Tenocytes	Human extensor carpi radialis brevis tendon	Improvement of patients' pain score by 86% after 12 months. Improved grip strength. Reduction in clinical prevalence of tendinosis. Functional improvement and structural repair of tendon.	Clinical trial evidence level 4	^35^
Tenocytes	Rabbit Achilles tendon	Increased collagen type I expression, demonstrating enhanced alignment. Increased stiffness of tissue.	*In vivo*	^102^
Dermal Fibroblast	Human patella refractory tendinopathy	Pain, severity, and functionality scores improved from 44 to 75 after 6 months. Decrease in tendon thickness.	Randomized controlled trial; level of evidence, 1	^49^
Muscle-derived stem cells	Mouse muscularis fascia of dorsum	Formation of cord-like neo-tendon similar to native tissue in appearance. Increased maximum load capacity. Increased stiffness at 12 weeks. Increased tensile strength.	*In vivo*	^102^
Tendon stem cells	Rat patella tendon	Increased expression of collagen type I and III, and tenomodulin. Formation of tendon-like tissue after 8 weeks. Enhanced collagen fiber thickness.	*In vivo*/*in vitro*	^46^
Fibroblast	Rabbit infraspinatus tendons	Increased type I collagen expression. Increased tensile strength of regenerated tissue.	*In vitro*	^103^
Periosteal progenitor cells	Rabbit infraspinatus tendon	Increased matrix deposition. Increased production of aggrecan and collagen type I and II. Formation of fibrocartilage and bone at the tendon–bone insertion site.	*In vivo*	^102^

ADSC, adipose-derived stem cells; BM-MSC, bone marrow-derived mesenchymal stem cell; GAG, glycosaminoglycan.

One of the biggest issues surrounding the use of cells for regenerative therapeutic is finding the most appropriate source, each having their own specific limitations,^2,17^ and currently there are no standard cell culture conditions available, meaning that *in vitro* and *in vivo* conditions used always differ. Tenocytes are the predominant cells in tendon tissue that have been studied extensively. The major issue surrounding the use of tenocytes is that they have a very limited availability, and the use of allografts can lead to morbidity at the donor site.^55^ After harvesting, issues can then arise in cultures with multiple passaging, leading to a loss in tenogenic markers, phenotypic drift, and a reduction in metabolic activity.^2^ TSC also have limited availability and are susceptible to a loss of phenotype during expansion; currently, these cells have not managed to show enough potential to warrant large preclinical animal model investigation. Similarly, the implantation of TSC alone only led to minor tendinous tissue formation.^46^ The use of nontendinous cell sources such as dermal fibroblasts or muscle cells can often result in complications and concerns surrounding tissue specificity. The use of stem cells has been linked to ectopic bone formation; they require specific tenogenic differentiation *in vitro* before use, which can often be complicated. In addition, multiple cultures can result in the loss of stemness. Currently, there is a lack in a panel of tendon-specific markers for identification. Characterization usually relies on the presence of a whole host of transcription factors related to tendon differentiation, but lacking specificity such as scleraxis, or ECM proteins such as collagens and tenomodulin.^2^ For example, TSC express the same panel of markers needed to characterize MSCs according to the international society for cellular therapy,^56^ and many factors can influence the expression of these markers, including age of the cell source, the donor tendon, and culture protocols.^15^ The exact mechanism of ADSC is unknown, and it, thus, presents the risk of unpredicted hazards if used in a clinical setting. In addition, studies have shown that increases in aggrecan that are seen suggest that these cells differentiate toward a more chondrogenic linage. The use of cells for the regeneration of tendon tissue is also extremely cost intensive, and current clinical trials show either low levels of evidence or only minor benefits.

## Proteins-Based Therapy in Tendon Regeneration

One of the potential therapeutic approaches emerging for the regeneration of tendon is the sustained release of cytokines and growth factors.^57,58^ The use of exogenous growth factors presents the possibility of accelerating cell proliferation, collagen synthesis, and extracellular matrix synthesis, leading to quicker recovery and enhanced repair.^1,34,59^ Several growth factors are currently being studied for this purpose (Table 2). These include insulin-like growth factor, platelet-derived growth factor, basic fibroblastic growth factor, bone morphogenetic proteins (BMP), transforming growth factor beta (TGF-β), and vascular endothelial growth factor (VEGF).^23^ All have been shown to be expressed and are important in nearly every phase of healing progression.^33,60^ VEGF-111 was evaluated for its potential to enhance tendon healing in rat Achilles tendon. A local injection of VEGF-111 resulted in an increased force to failure and ultimate tensile strength from a single dose.^61^ Furthermore, the continuous slow release of VEGF caused increased vascularization, resulting in accelerated healing of the tendon-to-bone insertion site of patients with rotator cuff injuries.^62^ VEGF is well known to stimulate angiogenesis.^63,64^ It is important in the early phases of tendon healing and it increases the vascularity of tissue and the corresponding proliferation of endothelial cells.^21^ In acute tendinopathy of patella tendons, patients exhibited a higher VEGF expression when compared with those suffering from the chronic condition. This has led to the suggestion that an increase in VEGF expression may lead to an accelerated healing after acute injury, especially when mechanical load is kept to a minimum.^64^ Current data suggest that VEGF has the potential to increase the tensile strength of tendon and to augment the healing process.^65^ However, contradictory research exists that questions whether increased vascularization is, indeed, beneficial. It has been suggested that a relationship may exist between neovascularization and pain in Achilles tendon. Areas of increased pain and palpable tenderness are heavily correlated with neovasculature within the tissue, suggesting that an increase in vascularization would, in fact, be detrimental.^66^ It is also possible that increased vascularization is limited in its application to early tendon healing alone, with insufficient numbers of studies and clinical trials being carried out thus far.^62^ Correspondingly, the current opinion of the benefit of VEGF as a therapeutic agent for the regeneration of tendon tissue is still inconclusive, requiring further investigation and evidence of improved clinical outcomes. When TGF-β3 was delivered to the repair site of supraspinatus tendon-to-bone insertions of rats, cell proliferation, vascularity, and an accelerated healing process were observed. Blind evaluation indicated an increase in cellularity at 14 days of tendency toward a stiffer tissue, an increase in ultimate strength, and a decreased cross-sectional area. The results of this study demonstrated the ability of TGF-β3 to significantly increase the biomechanical and structural properties of the tendon, leading to a better quality tissue when compared with controls.^67,68^

**Table 2.** Current Strategies for the Delivery of Exogenous Growth Factors for the Regeneration of Tendon Tissue *In Vivo*

*Treatment*	*Tendon model*	*Results*	*Ref.*
PDGF-BB	Canine flexor tendon	Increased cell density and proliferation. Increased expression of collagen type I. Thirty percent increase in reducible crosslinks.	^104^
PRP	Equine superficial digital flexor tendons	Increased cellularity. Increased collagen and GAG content. Increased tensile ability. Increased collagen matrix integrity.	^74^
VEGF-11	Rat Achilles tendon	Increased ultimate tensile strength of tendon. Increase in mechanical stress needed to rupture healed VEGF tendons compared with controls.	^61^
IGF-1 and TGF-β	Rabbit patellar tendon	Increased vessel formation. Production of fibrous repair tissue, with enhanced orientation. Increased force at failure, ultimate stress and stiffness at 2 weeks.	^68^
BMP-12 protein	Sprague–Dawley rats calcaneal tendon	Increased expression of tenocyte lineage markers such as Scx and Tnmd. Formation of tendon-like tissue. Increased cell proliferation. Elongation and alignment of cells, and increased matrix deposition.	^105^
BFGF	Rat Rotator cuff tendon	Increased production of GAG. Improved collagen organization, stiffness, and ultimate load to failure 8 weeks postoperatively. Improved healing at enthesis.	^106^

A more extensive review of this is covered elsewhere.^4^

BFGF, basic fibroblastic growth factor; BMP, bone morphogenetic protein; PRP, platelet-rich plasma; VEGF, vascular endothelial growth factor.

However, as with the delivery of cells, therapies requiring the delivery of proteins have their own limitations. Problems are often encountered when attempting to deliver the therapeutic agent to the target site, as they need to escape degradation that is long enough to be effective. Current applications for growth factors are limited by the short half-lives of these molecules,^1,21^ and by the lack of protein retention at the repair site.^69^ This highlights the need for an effective delivery system that is able to retain growth factors and cells at the site of injury, with continued controlled release, and allow the sequential administration of different factors. A suitable delivery system will offer the benefit of lowering the effective treatment dosage as well as of reducing the number of injections necessary for positive outcomes.^21,70^

The optimum delivery platform for proteins and growth factors is yet to be identified. Recombinant growth factors have a very short half-life when in physiological conditions, and they are quickly cleared from the site of injection. This means that very high doses are required to be clinically efficacious or multiple injections are needed, leading to high costs and encumbrance to patients.^21^ One of the biggest questions researchers must answer is what is the safest and most efficacious does, and this will change depending on the number and type of growth factor being employed in each formulation.^70^ Currently, the use of growth factors sparks controversy within the field, with bodies of research both advocating and refuting their use. Each deliverable factor, although with its advantages, is not without limitations. For example, the use of PRP is surrounded by a large amount of controversy within the field, with several newer studies suggesting that it has little to no benefit when compared with saline injections alone. A recent double-blind randomized clinical trial where patients were given ultrasound guided injections of either PRP or saline revealed that even after follow-up at 1 year no significant differences were seen between the two groups.^71^ In addition, there is currently some concern over the potential for PRP to secrete inflammatory mediators, which would have a negative impact on healing.^64^ Complications also arise when considering the reproducibility of results; due to the high diversity seen between each batch of PRP, more work is needed to ascertain the most efficacious production protocol.^59^ One of the possible explanations for this inconsistency seen in the application of growth factors is that *in vitro* results are not successfully translated to improvements *in vivo.*^34^

Furthermore, most studies into the benefit of injectable growth factors have employed the use of a single factor and, as such, there is a large range of controversy with variable results. The use of a single factor may prove to grossly oversimply the complex processes needed to fully stimulate the healing of tendon tissue.^63^ Considering the complex cascade of events that occurs during the healing of tendon tissue, it is likely that a multitude of factors will need to be applied at one time, the optimum cocktail has not yet been identified, and a multitude of possibilities exist.^65^

Considering that tendon repair is a lengthy process and the final stages are often not reached until after a year after injury, animal studies have a limited duration. In addition, due to the nature of tendon injury, a wide variety of lesions are likely to occur in patients that are not recreated during *in vitro* testing, with most injuries being due to direct surgical input or via enzymatic degradation, which does not successfully represent real injury.^72^

## Gene-Based Therapy in Tendon Regeneration

Gene delivery is designed to supply exogenous genetic materials into cells to alter the DNA and to induce, silence, upregulate, or downregulate the expression profile and secretion of proteins.^73,74^ Gene delivery has the advantage of resulting in the production of proteins that are synthesized naturally by host cell mechanisms, and, as such, is not associated with reduced bioactivity and activation of an immune response, which is often encountered when delivering exogenous biomolecules.^4^ In addition, gene delivery provides the opportunity for long-term protein release and availability, reducing the need for multiple injections of external factors and excessive therapeutic dosages. However, despite these advantages, gene delivery is limited by the susceptibility of nucleic acids to degradation and attack by immunocytes. If not sufficiently protected, naked nucleic acids are quickly destroyed and do not survive extended periods of time in plasma.^75^ This coupled with the challenges associated with efficient uptake of nucleic acids into host cells make it necessary for a suitable delivery system to be used for the effective delivery of small interfering RNA.

In the past few years, several studies have focused on establishing gene delivery systems for the healing and regeneration of tendon tissue (see Table 3). Plasmid DNA-encoding fibromodulin with histidylated liposomes and histidylated liner polyethylenimine polycomplexes were transfected by using a rat Achilles tendon laceration model. The results showed that, based on stiffness and histological analysis, treated tendons demonstrated an enhanced healing response that resulted in a tissue that was more phenotypically similar to healthy tendon than control groups.^76^ In a further study, adenovirus promoting the expression of BMP-14 was transfected into the rat Achilles tendon laceration model. Histological and biomechanical effects were examined, and results showed that tendon treated with transfection exhibited 70% greater tensile strength and increased cellular proliferation of tenocytes when compared with control groups at 2 weeks postrepair. No inflammatory response or production of undesired bone or cartilage formation was observed as an effect of adenovirus.^77^

**Table 3.** Genes Related to Tendon Regeneration and Their Function^4,107–109^

*Gene symbol*	*Corresponding molecule*	*Primary function*	*Ref.*
*COL1A1*	Collagen type I	Extracellular matrix structural constituent synthesis; identical protein binding.	^107,110^
*COL3A1*	Collagen type III	Extracellular matrix structural constituent synthesis; identical protein binding.	^12,107^
*COL5A1*	Collagen type V	Extracellular matrix structural constituent synthesis; identical protein binding.	^111,112^
*TNC*	Tenascin-C	Encodes an extracellular matrix protein.	^5,107^
*ACAN*	Aggrecan	Encodes an extra cellular matrix protein; mutations in this gene may be involved in skeletal degeneration.	^5,12^
*MMP*	Matrix metallo-proteinase	Proteins of the MMP family are involved in the hydrolysis of extracellular matrix in healthy tissue.	^4^
*TGFB1*	Transforming growth factor beta 1	Encodes a member of the TGF-β family of cytokines.	^113,114^
*IGF-1/IGF-2*	Insulin-like growth factor	Encodes IGF-1/IGF-2, which is processed from a precursor, bound to a receptor, and then secreted.	^115^
*PDGFA/PDGFB*	Platelet-derived growth factor alpha/beta	Encodes PDGFA/PDGFB.	^116^
*ELN*	Elastin	Encodes elastin, an extracellular matrix structural constituent.	^108,109^
*FBN2*	Fibrillin 2	Encodes fibrillin 2, an extracellular matrix structural constituent.	^108,109^
*LAMA4*	Laminin alpha 4	Encodes laminin alpha 4, a family of extracellular matrix glycoproteins, which are the major non-collagenous constituents of basement membranes.	^108^
*Scx*	Scleraxis	This gene encodes a protein that is expressed during embryonic development of tendons and ligaments.	^117,118^
*Tnmd*	Tenomodulin	This gene is also a candidate gene for age-related macular degeneration, though a direct link has yet to be demonstrated.	^118,119^
*Fmod*	Fibromodulin	The encoded protein may play a role in the formation of extracellular matrix and also regulate TGF-β level.	^78^

Gene delivery systems have also been assessed for their ability to downregulate the expression of proteins and to reduce adverse effects of tendon healing such as the formation of scar tissue (see Table 4). Rat patellar tendon cells were transfected with lentiviral-encoded shRNA (small hairpin RNA) to specifically silence the expression of decorin. Histological and biomechanical studies showed that downregulation of decorin to an appropriate level can promote the repair and regeneration of patellar tendon, and it can result in a reduction in scar formation.^78^ Downregulation of the intra-synthesis of TGF-β1 can be beneficial for the regeneration of tendon tissue. A poly lactic-*co*-glycolic acid (PLGA) nanosphere delivery system that can incorporate plasmids was shown to specifically inhibit the expression of TGF-β1. The PLGA nanospheres were able to effectively deliver the plasmid into tenocytes and to inhibit the expression of TGF-β1, with an effect lasting more than 6 weeks. This resulted in an improved tendon healing.^79^

**Table 4.** Use of Gene Transfection as an Advanced Delivery System for Tendon Healing and Corresponding Vectors

*Gene*	*Vector*	*Delivery mode*	*Animal model*	*Ref.*
BMP-2, Smad8	Liposome	*In vitro*	Rat, Achilles	^120^
BMP-14	Adenovirus	*In vivo*/*in vitro*	Rat, Achilles	^79^
TGF-β1	Nanospheres	*In vivo*/*in vitro*	Chicken, flexor tendon	^114^
Gal	HVJ-liposomes	*In vivo*/*in vitro*	Rat, patellar	^121^
Lac Z	Adenovirus	*In vitro*	Rat Achilles, human rotator cuff	^82^

HVJ, hemagglutinating virus of Japan.

Adenovirus-mediated gene transfer has been studied in healing rat Achilles tendon and in human rotator cuff tendon cells in which the adenovirus vector resulted in successful transfection of LaCZ gene into tendon cells. Similarly, adenovirus vectors have been used for the delivery of exogenous FAK gene and BMP-12 gene into chicken tendon cells, resulting in a significant increase in expression of these genes when compared with controls, and in an enhanced healing of tendon tissue.^80,81^ Abbah *et al.* conducted studies by using plasmid DNAs encoding decorin and interleukin-10 to co-transfect human tenocytes using polyplexes to suppress the expression of TGF-β and demonstrated positive results *in vitro.*^82^ Delalande *et al.* used liposomes and polymersome as delivery vectors and demonstrated improved mechanical tendon strength after transfecting with plasmid DNA encoding fibromodulin in an *in vivo* model.^78^

Although gene-based therapy has shown great potential in treating tendon injury and degenerative conditions, however, there are still concerns regarding the safety profile of genetic materials, such as potential mutagenicity associated with the use of plasmids.^83^ On the other hand, the use of viral vectors is problematic and has been known to cause a serious immune response. On the contrary, the nonviral vectors are reported to have a safer profile but have a diminished transfecting capacity.^84^ Further work needs to be carried out to advance the current field toward developing more effective transfection materials with either no or minimal toxicity.

## Clinical Perspective and Delivery Platforms for Tendon Tissue Regeneration

**FIG. 2.**
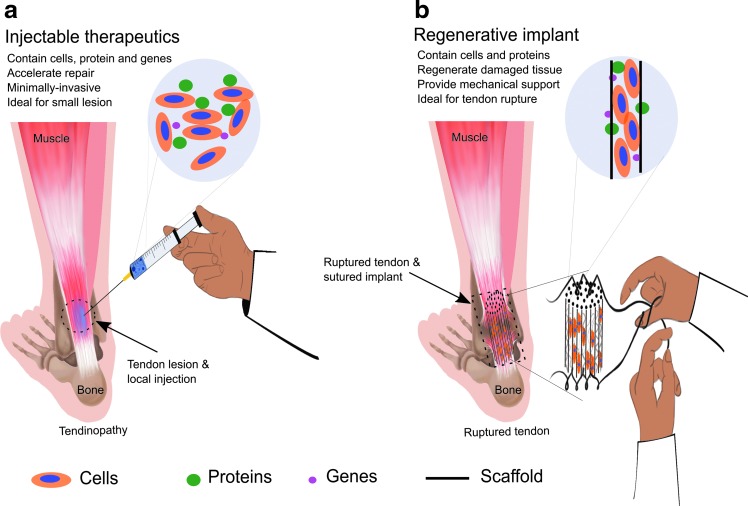
Tissue engineering strategies for tendon regeneration, including **(a)** injectable therapeutics containing cells, proteins, or genes, which can be directly injected to the site of the injury. **(b)** Regenerative implants containing a combination of cells, protein, and scaffold materials, which can be directly implanted and sutured in tendon rupture injuries.

To mimic successfully the spatiotemporal signaling profile seen in the healing process of tendon tissue, suitable biomaterials are needed to act as a delivery and supporting template for the sustained release of proteins, genes, and cells^85^ and to provide an architecture for cells moving into the site of damage.^20,86^ Current tendon tissue regeneration strategies are highlighted in Figure 2. Scaffolds can be used either as mechanical support or as carriers for deliverable factors. The main aim of the biomaterial scaffold is to provide a suitable environment for the attachment, proliferation, and migration of cells and to provide a foundation for matrix remodeling and tissue regeneration.^41^ The ideal biomaterial will closely mimic the native ECM architecture and biomechanical properties of tendon tissue.^44^ The biomaterial may be designed to incorporate cell adhesion moieties that allow for interaction with the host tissue, as well as for containing enzymatically sensitive regions that enable the degradation of the scaffold to occur simultaneously with *de novo* tissue formation. In addition, the ideal biomaterial would easily incorporate morphogens, signaling molecules, and cells that are able to co-ordinate the native healing process that results in the regenerative repair of the tissue.^44^ Biomaterials can include either natural or synthetic polymers, with some of the most common being collagen, chitosan, gelatin, alginate, hyaluronic acid, polyethylene glycol, polycaprolactone (PCL), polyglycolide, and PLGA.^87^ The precise biomaterial used and the scaffold structure can be altered to meet the requirements of the tissue, the deliverable factor to be encapsulated, for example, cells, proteins, or genes, and the implantation method into the host tissue.

## Injectable Hydrogels Systems

Hydrogels have potential as a biomaterial scaffold for the delivery of regenerative morphogens. Their polymeric networks can be easily functionalized and manipulated to have desired properties such as easy injection, mechanical stiffness and strength, and a controllable degradation rate.^88^ The polymer networks are hydrophilic with large water-absorbing capacity, and so are both biocompatible and able to closely mimic tendon ECM. They can be stabilized by using a number of crosslinking techniques or designed to be temperature or pH sensitive.

Hydrogels manipulated to encapsulate proteins and cells for their controlled delivery have been investigated in tendon models.^51,89^ An injectable poly (ethylene glycol) diacrylate hydrogel system was used for the delivery of BMP-2 protein and periosteal progenitor cells to defects at the tendon-to-bone interface of rabbit infraspinatus tendon. The hydrogel was able to fill successfully the defect, and the controlled release of regenerative factors resulted in the formation of fibrocartilage tissue around the tendon–bone interface after 4 weeks. Collagen type I production also increased around the tendon. Overall, the injectable hydrogel was able to increase the maturation and fibrocartilage formation around the tendon-to-bone interface, resulting in an improved healing response.^51^

Oligo(poly(ethylene glycol) fumarate) (OPF) and acrylated poly(ethylene glycol)–dithiothreitol (Ac PEG-DTT) hydrogels were produced with controlled degradation times ranging from a few days to 1 month. These were implanted into bovine patellar tendon defects *in vitro* to assess the ability of MSCs to infiltrate the area. It was found that by making these hydrogels degradable, the MSCs were able to infiltrate the defect site completely and concomitantly with degradation, whereas nondegradable structures resulted in MSCs concentrated around the sides of the defects. This suggests that hydrogels that can be controlled to degrade alongside the release of MSCs will result in the production of *de novo* tendon tissue that replaces the scaffold and fills the entire defect area.^89^

Hydrogel membranes consisting of xanthan gum, gellan gum, and hyaluronic acid were investigated for their ability to stop adhesion formation after tendon repair. The hydrogel membranes were placed into defects of the rat Achilles tendon, which were evaluated for histological and biomechanical strength after 3 weeks. The hydrogel membranes were able to degrade slowly and, thus, stayed present in the defect site during the entire period of healing. This resulted in much fewer adhesion formations and improved mechanical strength.^32^ In a similar study, collagen hydrogels were combined with electrospun fibers to produce a novel 3D hybridized collagen implant that was able to mimic tendon tissue in both its size and architecture. These implants were then used to fill Achilles tendon defects in 75 rabbits. When compared with controls, implants demonstrated a decrease in adhesion formation and muscle atrophy, and improved tissue alignment. In addition, the implant was fully degraded at the remodeling stage and was easily replaced with the newly formed tissue. Overall, the implant was able to produce an improved clinical condition, including tissue alignment and reduction of adhesions, within the animals.^90^ The ease of functionalizing hydrogels to have specific properties that can be manipulated to suit the demands of the healing tissue and deliverable factors is making them an increasingly popular choice of biomaterial for regenerative medicine.

Injectable strategies are advantageous for smaller tendon defects, benefiting as space-filling agents, as well as acting as a sealant and a barrier to the formation of adhesion sites.^32,91^ Their space-filling abilities allows them to be easily incorporated into irregular defects without the need for surgical intervention.^92^ They offer a simple and convenient method for the prolonged and controlled delivery of regenerative factors, and they allow for the proliferation and differentiation of cells that are necessary in the treatment of small defects.^93^ They offer a minimally invasive, convenient method for the prolonged delivery of cells and proteins to the defect site.^94^ Injectable systems have been advantageous in small lesions of the tissue and in ruptures where the torn end of the tissue proves difficult to re-suture, such as enthesis ruptures.

## Implantable Fibers System

Implantable materials are preferred in larger defects where the structural and mechanical properties of the tissue are greatly diminished.^93^ Implantable materials are able to bridge the gap created within larger midpoint ruptures, providing surgeons with the ability to remove the necrotic frayed end of the tissue, reconnect the tendon, and suture in place the regenerative device. This is particularly useful for full ruptures, and for partial ruptures that result in a large defect and a shortening of the tendon. Strategies that involve the use of a polymeric scaffold that is able to bear the mechanical loads subjected to the tissue while healing is taking place are more advantageous.^93^ Currently, implantable systems are more commonplace in tendon repair than injectable ones. Biomaterials can be woven or electrospun into fibers that are easily implanted into the defect site of tendon injury. Collagen has been extensively studied to assess its effectiveness as a biomaterial scaffold for the delivery of cells and proteins. When seeded onto electrochemically aligned collagen fibers, MSCs were able to differentiate down a tenogenic lineage *in vitro* and an increase in tendon specific markers such as scleraxis and tenomodulin was recorded.^94^ Collagen type I fiber scaffolds were shown *in vitro* to be conducive to a proliferative environment for tendon fibroblasts. It was found that by producing synthetic fascicle structures of collagen type I woven fibers, both tendon cells and white blood cells were able to infiltrate the entire scaffold and to adhere to the surface, with tendon tissue markers produced.^86^ In a similar study, collagen electrospun fiber implants, crosslinked via riboflavin and ultraviolet and combined with bovine platelet gel, were introduced to large Achilles tendon defects in rabbits. The biomaterial implant induced an enhanced inflammatory response, mediated by cells infiltrating the entire defect area and resulting in improved healing. The addition of the platelet gel to the collagen scaffold resulted in the increased proliferation and maturation of host fibroblastic cells, and, as such, increased the production of tendon matrix. Overall, the result was improved neo-tendon formation and healing. As the collagen implant degraded, the neo-tendon was able to form, was concentrated at the wound site, and was accompanied by a reduction in adhesion formations and muscle fibrosis.^95^

Other natural polymers have been investigated as scaffolds for tendon and ligament regeneration, including gelatin, fibrin, alginate, and chitosan.^44^ In an important study, electrospun and highly aligned chitosan fibers were produced and seeded with mesenchymal stem cells. Interactions with these aligned fibers resulted in a morphological change of the MSC toward a more fibroblastic phenotype. In addition, gene expression was altered, displaying a more tendon-specific profile, including a 50-fold increase in collagen type I expression. When these fiber scaffolds were implanted into rat Achilles tendon defects, it was found that this biomaterial together with the introduction of MSCs to the wound area resulted in an improved healing response when compared with randomly aligned fibers. Collagen expression, fibril diameter, stiffness, and force at failure were all increased in tendons in which aligned chitosan fibers were used.^96^

The use of synthetic polymers as biomaterials has also been investigated for the repair of tendon injuries.^97^ PGA/PLA fibers were used as a scaffold for the implantation of ADSC for the treatment of rabbit Achilles tendon, with surgically created defects that were 3 cm in length. Scaffolds consisted of an inner section of PGA fibers aligned longitudinally, and an outer layer of knitted PGA and PLA fibers to form a net-like structure. After *in vitro* expansion, ADSC were evenly seeded onto fiber scaffolds. Animals were sacrificed at 12, 21, and 45 weeks postsurgery. It was demonstrated that the longer the scaffolds were available at the repair site, the more improvement was seen, including the formation of neo-tendon tissue. A histological examination of neo-tendon confirmed similarities to native tissue, with an increase in collagen alignment, fibril diameter, and tensile strength. Tensile strength in the treatment group reached 60% compared with normal tendon, whereas control groups remained at 23%. In addition, cell-free control groups exhibited scaffold products at the repair sites at 45 weeks, fibrotic tissue, disorganized collagen, and increased inflammatory cells.^55^

Similarly, PGA electrospun fibers were used to deliver and to assess the ability of muscle-derived cells from mice to promote the formation of neo-tendon compared with tenocytes as a control. Cells were seeded onto PGA fibers and sutured to defects. Muscle cells were able to produce stronger tendon tissue, with thicker collagen fibers exhibiting increased maturity when compared with tenocyte-generated tissue. After 12 weeks, the cells had lost expression of muscle-specific markers such as MyoD and had an increased expression of tendon-specific markers.^98^ PCL electrospun fibers have been used in conjunction with silk fibroin yarns. The PCL is able to provide mechanical strength to the scaffold, and the silk fibroin yarns contain the crimp structure that is characteristic of tendon tissue. Fibroblasts grown in the presence of these constructs were able to proliferate at increased rates compared with controls, highlighting the ability of these biomaterials to promote and support cell growth.^99^ Fiber biomaterials are increasing in popularity due to their relative ease of suture to the repair site, and their ability to incorporate deliverable factors into prealigned structures resembling native tendon tissue.

## Allograft Decellularized Tissue

Decellularized tendon is emerging as a promising biomaterial for tissue regeneration. Decellularized tissue has the ability to exactly resemble the structure of tendon tissue while being able to provide the appropriate adhesion and signaling cues to host cells.^100^ As a scaffold, the decellularized tissue is able to allow the growth of cells along the aligned collagen fibrils present to mimic exactly the ECM present in healthy tendon tissue, leading to an improved healing response.^101^ Engineered tendon matrix from decellularized tissue was seeded with TSC from rat patellar tendons. This resulted in the production of tendon-like tissue after 8 weeks, with highly organized collagen fibrils. Collagen type I was extensively produced by these cells and overall, the result was the formation of neo-tendon that was comparable to that of healthy tissue.^46^ Similarly, the canine decellularized tendon tissue matrix was seeded with BM-MSC and tendon-derived stem cells (TDSCs). TDSC-seeded DTT produced significantly more collagen type I after 14 days than TDSC alone. The DTT scaffolds were able to produce tenogenic differentiation in both BM-MSCs and TDSCs, as well as to control the alignment of these cells.^100^ BM-MSC seeded onto decellularized tendon matrix and implanted in rabbit patellar tendon defects were able to demonstrate the cells' ability to proliferate and differentiate toward a tendon-like phenotype *in vivo*, with increased gene expression of tendon-related genes such as tenomodulin, collagen type III, and MMPs.^101^

## Conclusions

Treatment of tendon injuries and tendinopathies currently remains a challenge, with repair often resulting in the production of inferior tissue and long-term complications and morbidity for patients. The need for a treatment strategy that addresses the underlying pathophysiology of the damaged tissue is evident. This article highlights the recent developments in the field of biomaterials science for the application and delivery of regenerative factors for the repair of tendon tissue. There is an unmet clinical need for an advanced therapeutic that is able to recapitulate the spatiotemporal signaling pathways seen in the healing process of tendon tissue. The formulation of a therapeutic that is aimed at delivery of cells, proteins, and genes in a suitable biomaterial carrier may prove more successful than current singular approaches. The ideal formulation will be easily delivered to the site of repair, will degrade at a rate concomitant with *de novo* tissue formation, and will be able to stimulate the body's natural repair pathways, modulating cell proliferation and gene expression for the synthesis of essential tendon components. Further investigation is required to determine the ideal biomaterial that will be reproducible, scalable, nontoxic, nonimmunogenic, and bioresobable, with the ability to deliver spatiotemporal cues for the regeneration of tendon tissue. Current strategies based around either injectable or implantable systems are proving promising. Future exploration should be focused around the discovery of the optimum combination of cells, proteins, genes, and scaffolds that is able to orchestrate the complex chain of events leading to a regenerated tissue mimicking the native predamaged tendon, rather than the characteristic inferior scar tissue currently associated with repair.
